# Quality Controls: The Role of Self‐Corrective Science in Explorations of Primate Memory Systems

**DOI:** 10.1002/hipo.23667

**Published:** 2024-12-10

**Authors:** Elisabeth A. Murray

**Affiliations:** ^1^ Section on the Neurobiology of Learning and Memory National Institute of Mental Health, National Institutes of Health Bethesda Maryland USA

**Keywords:** amygdala, entorhinal cortex, hippocampus, macaque, medial temporal lobe, memory system, perirhinal cortex

## Abstract

In 1978, Mort Mishkin published a landmark paper describing a monkey model of H.M.'s dense, global amnesia. It depended on a combined removal of the amygdala and hippocampus (the A + H lesion) and a memory test called delayed nonmatching‐to‐sample (DNMS). My first project examined whether the impairment Mishkin had found in visual memory generalized to tactual stimuli. However, to gain access to the hippocampus and amygdala with 1980s surgical methods, we had to remove the underlying cortex. When we were able to test the effects of bilateral removal of that underlying cortex (the entorhinal and perirhinal cortex, or “rhinal cortex” for short) we obtained a dramatic result. This so‐called “control” lesion caused a profound impairment on the DNMS task. A few years later, excitotoxic A + H lesions, which left the rhinal cortex intact, confirmed that removal of the cortical “impediments” had caused the entire memory impairment that Mishkin had observed. These results: (1) forced a reconsideration of the monkey model of global anterograde amnesia; (2) spurred study of the independent contributions of the amygdala, hippocampus, and perirhinal cortex to cognition; and (3) led to the realization that the DNMS task did not test the kinds of memory that H.M. lost after his surgery.

The invitation to contribute an autobiographical account of my early neuropsychological studies evoked a flood of participatory memories about our crucial “control” experiments, which means that my hippocampus must have been functioning reasonably well back then. As we now know, the hippocampus is necessary for the establishment of explicit participatory memories: the stuff of autobiography. Naturally, I jumped at the chance to retrieve and reconsolidate these memories, as well as to tell the next generation of neuroscientists about how the dogged pursuit of a control experiment, which we initially expected to yield nothing of great interest in itself, led to most of what I have subsequently done. This article tells the story of that control procedure, our initial failure to do it right, and our try‐try‐again success that overturned the theory of memory systems that prevailed at the time and led to a new one.

## 1 Setting the Stage

I came to the Laboratory of Neuropsychology (LN) at the National Institute of Mental Health (NIMH) as a somatosensory specialist. Prior to that I attended Bucknell University, where I obtained a BS in Biology, and then the University of Texas Medical Branch at Galveston, where I earned a PhD in Physiology. For my dissertation, I examined the organization of corticospinal neurons in macaque monkeys. Since college, however, I had been interested in the neural bases of behavior, and after my graduate work I had a chance to pursue that line of investigation. My first choice as a mentor was Josephine Semmes, a neuropsychologist in the LN who studied the somatosensory cortex of macaques. Unfortunately, she had just retired. I soon learned, however, that Mortimer (Mort) Mishkin, a neuropsychologist in the LN who studied visual sensory processing and memory, might have a position opening. In due course, I applied for and obtained an NINDS postdoctoral fellowship to study somatosensory memory in his laboratory. After finishing my first series of studies, which explored the neural basis of tactual and crossmodal learning and memory (Murray and Mishkin 1983, 1984a, 1984b, 1985), I transitioned to a Staff Fellow position, a junior faculty appointment roughly equivalent to an assistant professor in a university setting. Around the same time, I convinced Mort to allow me to study visual memory. Somatosensory mechanisms of learning and memory were of interest to me then, and remain so, but they are much more difficult to investigate than visual memory, and such projects take much longer to complete. My 3 years as a postdoctoral fellow were amazing. Given my background in biology and physiology, I had so much to learn! The exposure to a stream of eminent visitors—including Brenda Milner, Larry Squire, Malcolm Brown, Trevor Robbins, Karl Pribram, David Premack, and Charlie Gross among many, many others—was deeply inspiring. And I loved working with monkeys. Rhesus monkeys ( *Macaca mulatta* ) and long‐tailed monkeys ( *Macaca fascicularis* ) were, and still are, endlessly fascinating. Not only are they outstanding models for studying human memory, but they are interesting in their own right. As ecological generalists, which establish cohesive societies, develop rapidly, and reproduce at a high rate with short intergenerational intervals, they are remarkably resilient and successful species. Except for the coldest climates, they can probably thrive just about anywhere, including the laboratory.

I was fortunate to enter the LN just as studies in monkeys became pivotal for understanding the neural substrates of memory. In 1979, when I joined the laboratory, studies of the clinical case known as H.M., who we now know as Henry Molaison, had gained renewed importance. As is well known, he developed a dense, global, anterograde amnesia following bilateral medial temporal lobe (MTL) removals for relief of epilepsy. His case presented a puzzle, one at the top of the list of many neuropsychologists at the time. What brain structures, when damaged, led to his severe, yet selective, memory impairment?

The original account by Scoville and Milner (1957) described that in the 10 patients studied, including H.M., the severe anterograde memory impairments occurred only after bilateral MTL excisions that included much of the amygdala, hippocampus, and surrounding cortex. That the deficit was specific to memory, and that other domains of cognition—including language and working memory—were intact, made these clinical cases all the more striking and informative. A monkey model of H.M.'s amnesia was an obvious goal, but the first attempts to establish one floundered, in part because they relied on the acquisition and retention of visual object discriminations (for review see Murray (1996)). The typical object discrimination problem involved two objects presented to the monkey on a two‐well test tray, again and again, for 30 trials per day. Each presentation was a ‘trial’ in which the monkey had a chance to earn food reward by displacing one of the two objects positioned over the food wells—one on the left and one on the right side of the test tray. One object was arbitrarily assigned the S+ (rewarded) and the other object the S− (unrewarded). The reward assignment was fixed within and across sessions; to ensure the monkeys' choices were based on object features, as opposed to position on the test tray, the location of the correct object (left or right) followed a pseudorandom order. Through trial and error, the monkeys learned to approach and displace the S+ to obtain the food reward hidden underneath. Displacing the S+ for 27 or more out of 30 consecutive trials for one or two consecutive days (or similar performance depending on the study) was taken as evidence that the monkey could visually discriminate the two objects, and could remember which one covered the reward. Monkeys with lesions that were intended to mimic H.M.'s were unimpaired on postoperative acquisition and retention of these memory tests. Both monkeys and humans can perform visual discrimination tasks in many ways, and it is likely that this is the reason for the long series of failures to produce a monkey model of H.M.'s amnesia.

After over a decade of failure, the 1970s saw the first glimmers of success. First, David Gaffan (1974) used a delayed matching task in which he required monkeys to remember single objects or a series (list) of objects over increasingly longer delay intervals. He employed a set of objects large enough to complete 5 days of testing without using any individual object more than once. Gaffan found that fornix transection impaired performance on the delayed matching task, especially in the conditions thought to tax memory the most. Then Mort and Jean Delacour found that monkeys performed well on a similar memory test that also used a large stimulus set. In addition, they found that intact monkeys learned the nonmatching rule faster than the matching rule (Mishkin and Delacour 1975), at least as the test was administered at the time. All four advances—lists of objects, trial‐unique objects, the nonmatching rule, and increasing delays between sample and test—were incorporated into later versions of the task. Although a matching‐to‐sample test had been in use for years, this new variant became standard, and the canonical delayed nonmatching‐to‐sample (DNMS) task was born.

**FIGURE 1:**
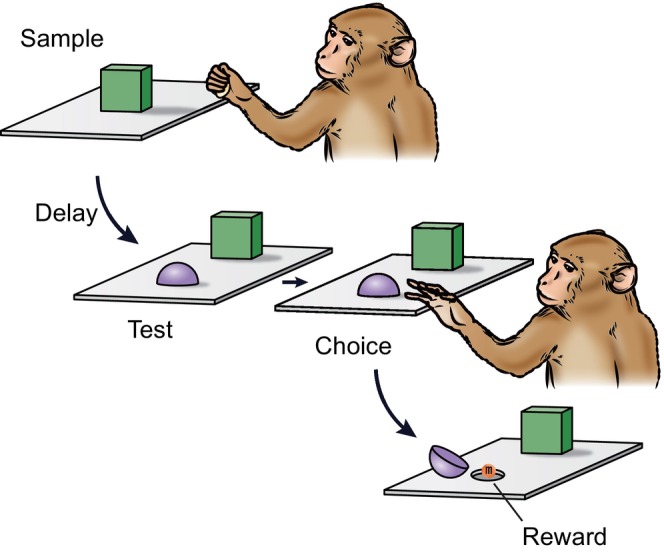
Schematic diagram of the DNMS task. Each trial is composed of two parts: sample phase followed by test phase. In the sample phase, monkeys displace a sample object to obtain a reward hidden underneath. After a delay, monkeys are presented with the sample object together with a novel object in the test phase. Monkeys could obtain an additional reward if they displaced the novel object. The task is typically administered with novel objects on each trial (e.g., trial‐unique objects).

From the 1960s through the 1990s, neuropsychologists studying nonhuman primates typically carried out DNMS testing manually in a Wisconsin General Test Apparatus (WGTA). As a postdoctoral fellow, I spent a fair amount of time conducting these procedures. First, I placed a sample object over the baited central well of a three‐well tray located between me and the monkeys. An opaque screen prevented the monkeys from seeing me or the tray at that time. In the sample phase of each trial, I raised the screen, and the monkeys could then see the sample object and obtain the food reward hidden underneath by displacing it. (A second opaque screen located between me and the test tray prevented the monkey from seeing me.) For the test phase of each trial, I placed the sample object in either the left or right well of the tray and a novel object on the opposite side, according to a pre‐arranged schedule. The monkeys could then obtain another food reward if they displaced the novel object, the one not matching the sample. If they displaced the object that had appeared as the sample, they got nothing. A schematic of the DNMS procedure is shown in Figure 1.

I first trained monkeys on the DNMS rule using short (10 s) delays between the sample phase and test phase. When they had learned the rule, and with no additional training, I gave them a performance test; I introduced delay intervals of 30, 60, and then 120 s between sample and test, or alternatively, presented a list of 3, 5, or 10 objects to remember. The performance test was thought to tax object recognition memory. The idea was that if monkeys remembered the sample object, they would choose the novel one during the choice test. The delays and lists were given in blocks of trials. For example, 5 days using a given delay interval (e.g., 30, 60 or 120 s) were administered before moving to the next longer delay interval, until the 120 s delay block was completed. A similar procedure was followed for the testing with lists of objects. Lists involved presentation of each sample in the list, one at a time, followed by the choice tests, one at a time, in the same order as the samples were given. (List‐length testing necessarily required increasing the delay between sample and test; accordingly, any effects of increasing the list length were confounded with increases in delay.) This constituted a ‘test’ in the sense that each trial offered the monkeys one chance to get the right answer. There was no correction for errors, and no attempt to train the monkeys with the challenging delay and list conditions.

Importantly, the label ‘object recognition memory’ glosses over an important question: How exactly did monkeys perform the task? Did they remember (and perhaps even rehearse) the sample? Did they simply learn to approach and displace novel objects, a strategy that was consistently rewarded during both the sample and choice phases of the task? Or did they track the relative recency of an object's presentation, based on how long ago, if ever, they had seen the two objects now presented in the choice phase? These questions are important because it eventually became clear that the label ‘object recognition memory’ had become a proxy term for a much more nuanced concept, most commonly called either declarative memory or explicit memory (Schacter 2024). Occasional denials notwithstanding, both terms imply conscious awareness of information stored in memory.

**FIGURE 2:**
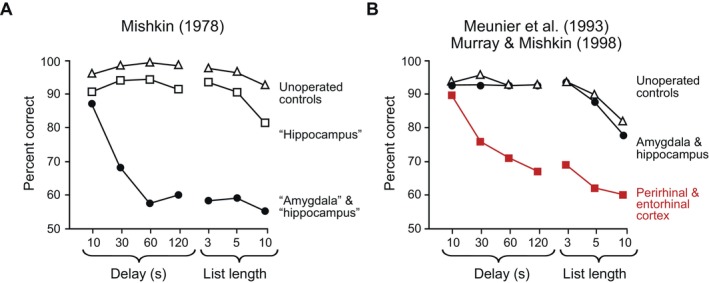
Performance on the delayed nonmatching‐to‐sample (DNMS) task. (A) Mishkin's results in unoperated control monkeys (triangles) and after what he called a hippocampus lesion (squares) and combined A + H lesion (circles). The names for these anatomical structures appear in quotation marks because they are not accurate descriptions of the actual lesion. (B) The results of our “control” experiments. Scores from groups of monkeys with bilateral removal of the entorhinal and perirhinal cortex (squares), along with unoperated controls (triangles) and selective, bilateral excitotoxic A + H lesions (circles). Adapted from figure 12.2 in E. A. Murray, S. P. Wise and K. S. Graham, The Evolution of Memory Systems, Oxford University Press, Oxford, 2017.

After the advances of Gaffan (1974) and Mishkin and Delacour (1975), Mort published a high‐profile article in *Nature* (Mishkin 1978). In a study of rhesus monkeys, he reported that combined but not separate damage to the hippocampus and amygdala led to severe impairments on the DNMS task (Figure 2A). In discussing this result, he suggested that it was combined damage to these regions that was likewise responsible for the severe anterograde impairment in declarative memory suffered by patient H.M. Importantly, monkeys with the A + H lesions performed well when there were short (10 s) delays between the sample and test phases, which showed that they knew the rules of the task, had the motivation to perform it, and could discriminate the objects. However, their scores fell dramatically with the imposition of longer delays or lists of objects. Thus, the impairment appeared to be specific to memory.

This landmark paper was followed by a review (Mishkin 1982) in which Mort outlined his idea that object recognition memory required two parallel cortico‐limbic‐thalamocortical circuits: One involving the amygdala and mediodorsal nucleus of the thalamus; and the other the hippocampus and anterior thalamic nuclei. Around the same time, Stuart Zola‐Morgan (now Zola) and Larry Squire initiated a series of studies to examine the neural substrates of memory in macaque monkeys. Although their initial studies examined the effects of combined amygdala and hippocampal removals (Zola‐Morgan and Squire 1985; Zola‐Morgan, Squire, and Mishkin 1982), later ones involved lesions limited to the hippocampus. Their results led them to conclude that the hippocampus was the critical structure for memory (Zola‐Morgan and Squire 1986; Zola‐Morgan, Squire, and Amaral 1989b). Thus, by the mid‐to‐late 1980s, two camps developed regarding the MTL structures critical for normal DNMS performance: (1) the hippocampus alone; and (2) the hippocampus and amygdala combined. Both ideas were wrong, but it was not until 1998—a full 20 years after Mishkin's report in *Nature*—that the requisite control experiments finally resolved the issue. In this article, I tell the story of how this exercise in self‐corrective science came to pass and how a junior scientist (me) came to perform experiments that overturned the cherished A + H theory.

## 2 The Discovery

Before relating the story in full, I'll briefly summarize what I see as our most important discoveries. Later, I'll spell out the results and their significance in more detail. The main advances for which I can claim some responsibility are as follows:

- First, based on neuropsychological studies in monkeys, we showed that the hippocampus was not essential for either visual or tactual object recognition memory as measured by the DNMS task (Murray and Mishkin 1983, 1984b). This finding confirmed for tactual stimuli what Mort had found for visual ones.
- Second, we showed that combined damage to the entorhinal and perirhinal cortex produced a severe impairment on the DNMS task (Meunier et al. 1993a).
- Third, we showed that combined, selective lesions of the amygdala and hippocampus made with the neurotoxin ibotenate had no effect on performance of the DNMS task (Murray and Mishkin 1998).

Together, these three findings, which were published over a period of 15 years (1983–1998), overturned the idea that either the hippocampus (alone) or the hippocampus and amygdala (combined) play a necessary role in performance of the DNMS task. If this task assessed object recognition memory, then neither the amygdala nor the hippocampus have this key cognitive function. But did the DNMS task really test that? I'll return to this question shortly.

## 3 The Road to Quality Controls

As noted above, my initial studies on tactual recognition memory—carried out as a postdoctoral fellow—were consistent with and extended the results Mort had reported in his landmark *Nature* paper: Combined damage to the amygdala and hippocampus led to severe impairments on both tactual and visual version of the DNMS task (Murray and Mishkin 1984b). H.M.'s impairment was global, which means that it included all sensory modalities, among other things. So, it was important to establish that the impairment extended beyond visual memory. Furthermore, at around the same time, Richard Saunders—during a brief stint as a research assistant in the LN before he departed for graduate school—found that damage to 3 of the 4 structures (either two amygdalae and one hippocampus, or one amygdala and two hippocampi) led to an impairment roughly halfway in magnitude between the impairment observed after bilateral removal of either the amygdala or hippocampus (2‐structure lesions) and the combined removal of both (a 4‐structure lesion) (Saunders, Murray, and Mishkin 1984). Results from other laboratories agreed with these findings (Zola‐Morgan and Squire 1985).

A fly in the ointment was that the interpretation of these findings was more difficult than it seemed to be at first glance. All the studies mentioned so far were based on aspiration lesions of the amygdala and hippocampus, structures deep within the MTL. Aspiration, which was the only reliable method of producing such a lesion at the time, included removal not only of the intended target structures, but also of the underlying entorhinal and parahippocampal cortex. In the initial experiments, these structures were viewed as impediments to gaining access to the amygdala and hippocampus. This interpretational limitation was always noted in the published reports, but without the emphasis that, in retrospect, it clearly deserved. The obvious control experiment was to remove the entorhinal and parahippocampal cortex, and, for reasons explained below, the perirhinal cortex, as well. To be fair, it simply was not feasible at that time to remove, by aspiration, the entire perirhinal, entorhinal and parahippocampal cortex. These structures are located in the extreme ventromedial part of the temporal lobe, and there was no surgical approach to remove them that would not also have damaged the amygdala and hippocampus. In my first major memory study (Murray and Mishkin 1984b), we noted that damage to the entorhinal cortex might be responsible for the DNMS impairment after so‐called A + H lesions. As we expressed this caveat at that time: “whereas amygdalectomy alone involved approximately the rostral one‐third of the entorhinal cortex and hippocampectomy alone involved approximately the caudal one‐third of the entorhinal cortex, the combined ablations of the amygdala and hippocampus destroyed all of it” (Malamut, Saunders, and Mishkin 1984, 2579). Because the entorhinal cortex, like the amygdala and hippocampus, possessed neuroanatomical connections with other regions implicated in memory, and could plausibly be responsible for the impairment, this alternative interpretation of our results needed to be addressed.

The way forward therefore required at least two more studies. First, it was important to evaluate the effects on recognition memory of entorhinal cortex lesions. Second, it seemed of equal importance to evaluate the effects on memory of combined, selective lesions of the amygdala and hippocampus.

### 3.1 Impediments and Controls

Because it was impractical to do the obvious control experiment of testing the effects of removal of the cortex subadjacent to the amygdala and hippocampus, we did the next best thing. We decided to study the effects of lesions of entorhinal cortex together with either the amygdala or the hippocampus. As a prelude to conducting a control experiment involving lesions of the entorhinal cortex, I surveyed the literature on anatomical connections of the region. It became apparent that many of the connections of the perirhinal cortex were similar to those of the neighboring entorhinal cortex. For example, both regions received projections from the inferior temporal visual cortex, and both projected into the medial portion of the mediodorsal nucleus of the thalamus. Although now it is well established that the perirhinal cortex is a multimodal region contributing to the representation of objects, at the time there was virtually nothing known about it. So, because we wanted to test the A + H model of amnesia, it seemed prudent to remove both the entorhinal cortex and perirhinal cortex in the planned control experiments. For convenience, we invented a name for this combination of cortical areas: rhinal cortex. That study (Murray and Mishkin 1986) yielded another result consistent with Mort's 1978 paper (Mishkin 1978). Specifically, we found that removals of the amygdala plus rhinal cortex produced a severe impairment on the DNMS task, one at least as severe as that observed after combined amygdala plus hippocampal lesions in Mort's original study. By contrast, removals of the hippocampus plus rhinal cortex (H + Rh) led to a relatively mild impairment. As it turned out, the latter result was highly misleading, due to sparing of rostral rhinal cortex in the H + Rh group. This error was corrected in due course (Meunier et al. 1993b, 1996). Fortunately, it did not set back our progress. Indeed, preliminary results from the next experiment (Murray, Bachevalier, and Mishkin 1989), described below, helped us move forward.

### 3.2 Entorhinal and Perirhinal Cortex

As mentioned above, the entorhinal and perirhinal cortex are located in the ventromedial aspect of the temporal lobe, its most inaccessible part. Fortunately, Jocelyne Bachevalier, a friend and colleague in the LN, had been studying the effects of occlusion of the posterior cerebral artery (Bachevalier and Mishkin 1989). To this end, she developed a neurosurgical procedure that had an unexpected feature: her surgical approach to the posterior cerebral artery provided access to the ventromedial temporal lobe. Tackling the problem together, we adapted the approach to gain access to the rhinal sulcus. With the aid of an operating microscope, we were able to identify and remove the entorhinal and perirhinal cortex using standard aspiration methods. Finally, we could now perform the control experiment I had always wanted to do, without involving the hippocampus or amygdala, at least not directly.

Around the same time, my first postdoctoral fellow, Martine Meunier, joined the laboratory. Martine, Jocelyne and I studied a group of unoperated control monkeys together with three experimental groups, all with bilaterally symmetrical lesions: (1) of the entorhinal cortex alone; (2) of the perirhinal cortex alone; and (3) of the entorhinal and perirhinal cortex combined, that is, the rhinal cortex (Meunier et al. 1993a). When they were tested on the DNMS task, we found that monkeys with entorhinal cortex lesions had a mild impairment, whereas those with perirhinal cortex lesions had a severe impairment. Remarkably, monkeys with rhinal cortex lesions (Figure 2B) were almost as impaired as the monkeys with A + H lesions in Mort's 1978 paper (Mishkin 1978) (Figure 2A). A study in David Gaffan's laboratory (not illustrated) replicated these findings (Eacott, Gaffan, and Murray 1994).

These findings surprised everyone. After all, these were control experiments meant to merely refine and improve the results we had reported earlier. The effects of perirhinal cortex lesions were especially unexpected. In my first study (Murray and Mishkin 1983, 1984b), we argued that the perirhinal cortex was unlikely to have contributed to the A + H results. This is because an examination of Nissl‐stained sections of the brains of each lesioned monkey, which I carried out as part of the process of reconstructing the lesions, indicated that damage to this cortical area was minor, confined to its posterior part, and asymmetrical in the two hemisphere. Because of our earlier results, it did not seem at all likely that it would overturn the A + H model of H.M.'s amnesia. It was perhaps for that reason that Mort permitted these experiments to go forward. I'm sure he had every expectation, as I did initially, that they would provide more evidence consistent with his view. Instead, we found that it was combined damage to the anterior and posterior parts of the rhinal cortex, rather than combined damage to the amygdala and hippocampus, that was responsible for the severe impairment caused by lesions we had called, misleadingly, A + H lesions. Figure 2A highlights this discrepancy by placing quotation marks around “amygdala” and “hippocampus”.

Despite all these results, we still did not understand how the surgical procedure Mort used to make A + H lesions had caused such a severe impairment. Our histological data showed that Mort's A + H lesion had left most of the perirhinal cortex intact. Furthermore, Mort's surgical approach to the A + H lesion, as for all other lesions, was extremely systematic. If cortex lateral to the rhinal sulcus was not part of the ‘intended lesion’ (it was not), then it was not removed. Why wasn't the intact perirhinal cortex sufficient for reasonably good performance on the DNMS task?

**FIGURE 3:**
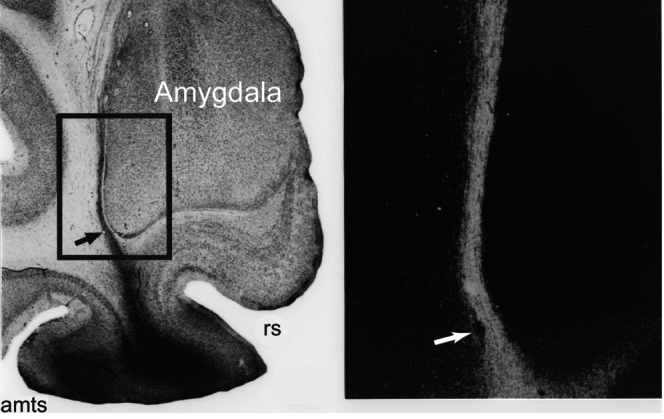
Autoradiograph of perirhinal cortex efferents. *Left*: Silver grains (black) reflect the transport of radiolabeled proteins in axons emanating from the perirhinal cortex. In this bright‐field photomicrograph of a Nissl‐stained coronal section, the square indicates the region of interest. amts, anterior medial temporal sulcus; rs, rhinal sulcus. *Right*: Dark‐field illumination of the region in the box to the left. Labeled axons appear light gray. Arrow: Blood vessel that provides a reference point in both parts of the figure. Medial, right; dorsal, up. Reproduced from E. A. Murray, Medial temporal lobe structures contributing to recognition memory: The amygdaloid complex versus the rhinal cortex. In The Amygdala: Neurobiological Aspects of Emotion, Memory, and Mental Dysfunction, pp. 453–470, 1992, CCC Republication.

Once we posed the question that way, it soon became apparent that the combined A + H lesions rendered the perirhinal cortex dysfunctional by damaging nearby fiber tracts, especially those coursing lateral to the amygdala. Figure 3 shows a photomicrograph of material from an anatomical tracer study carried out by Leslie Ungerleider, another friend and colleague in the LN. We had been discussing anatomical connections of the perirhinal cortex when she noted that she had an anatomical case I could examine. Upon examining the tissue slides under the microscope, I was instantly convinced they explained the profound impairment caused by aspiration lesions of the amygdala (when combined with other structures). If ever a picture is worth a thousand words, Figure 3 fits the bill. It was clear that aspiration lesions of the amygdala had inadvertently interrupted perirhinal efferent fibers laterally adjacent to the amygdala. This was the main reason why aspiration lesions that included the amygdala caused such a large impairment on the DNMS task (Murray 1992); the A + H removal included not only the amygdala and underlying entorhinal cortex, which was recognized at the time, but also rendered the adjacent perirhinal cortex dysfunctional. The stimulus material and testing procedures also influenced the results, but to a lesser extent. For example, if the stimuli used for testing had not been represented by the perirhinal cortex, cutting these fibers would have had no effect. But representing visual and tactual objects is precisely what the perirhinal cortex does.

Somewhat later, another postdoctoral fellow in my group, Sonia Goulet, together with visiting professor Francois Doré, tested the idea that amygdala aspiration lesions disrupted perirhinal efferent fibers. We did this by examining the integrity of corticothalamic projections in monkeys with aspiration removals of the amygdala. In brief, we injected retrograde tracers into the medial portion of the mediodorsal nucleus of the thalamus, bilaterally, in monkeys with unilateral aspiration removals of the amygdala, and then compared the numbers of retrogradely labeled cells in hemispheres with and without the lesion. The rhinal cortex of the amygdalectomized hemispheres consistently contained significantly fewer labeled cells relative to the rhinal cortex of intact hemispheres. A similar asymmetric pattern was observed for visual area TE but not for the cortex lining the dorsal bank of the superior temporal sulcus, nor for the rostral cingulate motor area, which we examined as control areas. We concluded that aspiration lesions of the amygdala not only removed the cell bodies of the amygdala, as intended, but also inadvertently transected projection fibers arising from cells in the rhinal cortex and neighboring area TE; the transected fibers were presumably those coursing nearby the amygdala en route to the thalamus, as shown in Figure 3 (Goulet, Dore, and Murray 1998) and undoubtedly affected many additional corticofugal targets, as well. Afferents to the rhinal cortex may also have been transected by aspiration lesions of the amygdala, although we did not examine this possibility.

### 3.3 Amygdala and Hippocampus

The second goal, to evaluate the effects on memory of combined, selective A + H lesions, had to await the technological advance of magnetic resonance imaging (MRI).^1^ Although excitotoxins were in wide use to make selective lesions, there was no reliable method of placing those injections in deep brain structures in macaques. A stereotaxic atlas of the macaque brain was available, but it was of little use for surgical planning because of the high individual variability of brain shape and size. For example, the anterior–posterior distance between the external auditory meatus (interaural plane, or ear bar 0) and the mid‐amygdala in rhesus monkeys can range from approximately 16 to 24 mm and is only weakly correlated with a monkey's weight. The distance from the mid‐amygdala to the sphenoid bone, however, ranges from roughly 3 to 5 mm and has a much stronger correlation with an animal's weight (Aggleton and Passingham 1981).

In the early 1980s, John Aggleton was also a postdoctoral fellow in the LN. During this period of overlap in our careers, he shared the method he and Dick Passingham had developed to localize the amygdala relative to bony landmarks identified in x‐ray images of the head (Aggleton and Passingham 1981). This gave me the opportunity to perform selective amygdala lesions. After obtaining x‐ray images of a monkey's head, I was able to calculate the location of the amygdala, and could use a stereotaxic approach to inject excitotoxins into my target. In a pilot study, I combined excitotoxic amygdala lesions with aspiration lesions of the hippocampus and then tested the monkey on the DNMS task. Contrary to the A + H model of amnesia, the animal was unimpaired despite the combined amygdala and hippocampal damage (personal observations). Clearly we needed to follow up this preliminary finding by conducting a proper study with a group of monkeys with selective lesions of the amygdala and hippocampus and concurrently tested controls, as described in the next paragraph. By that time, I had developed a healthy degree of humility about what was a “control” lesion and what was the principal lesion in neuropsychology experiments. The results of our rhinal cortex lesion seemed to account for the entire impairment, but this did not rule out an important contribution from the amygdala and/or hippocampus. Both the perirhinal and entorhinal cortex have dense, reciprocal connections with the amygdala and the hippocampus, and a few years earlier Squire and Zola‐Morgan (1991) had proposed that lesions of the entorhinal cortex rendered the hippocampus dysfunctional, in part due to a loss of afferents.

With the advent of MRI and the growing use of imaging in clinical settings, we could obtain structural scans of each monkey's head, which meant we could obtain a stereotaxic atlas for each subject. Richard Saunders, together with Tom Aigner (LN) and Joseph Frank, a radiologist in the NIH Clinical Center, showed this was possible if one used a nonferrous stereotaxic instrument (Saunders, Aigner, and Frank 1990). This is precisely what we needed to produce the combined A + H lesions: a method for the injection of excitotoxins based on MR‐guided stereotaxic approaches. Finally, we carried out the needed study. Unsurprisingly, to me at least, monkeys with combined A + H lesions were unimpaired on the DNMS task (Figure 2B) (Murray and Mishkin 1998). Postoperatively, monkeys with the combined, selective A + H lesions performed as well as unoperated controls at every stage of testing. In a special form of DNMS testing devised by David Gaffan (Eacott, Gaffan, and Murray 1994), we presented 40 objects sequentially as samples before any choice tests. The objects were presented one at a time, separated by 30s. Then, with 30‐s delays between choice tests, the sample objects appeared in reverse order: the last sample appeared in the first choice test and the first sample appeared in the last choice test. Unlike the standard list‐length tests described earlier, this procedure yielded a wide range of delay intervals without requiring the monkeys to sit through long unfilled intervals. By the time the first sample appeared in a choice test, approximately 40 min had elapsed. Even so, monkeys with the selective A + H lesions performed at the same level as unoperated control monkeys.

We carefully examined the extent of hippocampal cell loss and came up with another surprising finding. Monkeys with the most hippocampal damage had better memory scores than monkeys with less damage. This observation argued against the possibility that the lesions were ineffective and suggested that disabling more of the hippocampus diminished a counterproductive (probably spatial) strategy.

To provide some perspective, the results discussed above were almost all presented in preliminary form at Society for Neuroscience meetings. At that time, most of the meeting presentations were either major lectures, symposia talks, or 15‐min platform talks; compared to the present format, there were relatively few posters. Because of the intense interest revolving around H.M. and the neural substrates of memory, there was an annual session devoted to monkey memory, a session typically dominated by results emerging from Mort's and Larry's labs. I recall the excitement of presenting the ‘final’ neurotoxic A + H lesion results at the 1996 Society for Neuroscience meeting in Washington, D.C. (Murray and Mishkin 1996). The platform presentation was well received, and—thanks to having so many colleagues in attendance—it was a celebratory moment. Among my most ardent supporters and mentors in these relatively early days were David Gaffan, Lynn Nadel, Morris Moscovitch, Malcolm Brown, Sue Corkin, Trevor Robbins, Michela Gallager, Len Jarrard, Norman White, and David Olton. (Of course many more colleagues provided support at later stages of my career, and many of my peers, both inside and outside LN, were terrifically influential as well.)

Although it took 20 years to complete the journey, this story has a clear message. Within the MTL, damage to the rhinal cortex is both necessary and sufficient to produce profound impairment in performance of the DNMS task. However, the relationship between these findings and memory systems in monkeys and humans, including H.M., is a longer story, one my colleagues and I have elaborated twice at book length, once for specialists in memory research (Murray, Wise, and Graham 2017) and a second time for general readers (Murray et al. 2020). In the closing section, I'll return to this.

## 4 Missing Pieces

In providing this first person historical perspective, an intentionally superficial account at that, it is important to provide some balance. We conducted our studies with the knowledge of what others in the field were thinking and doing at that time. Stuart Zola and Larry Squire—located at the University of California, San Diego—were also studying the neural bases for recognition memory in macaques, and were also interested in understanding H.M.'s amnesia. Results emerging from one laboratory often spurred a study in the other.

One set of findings relates to the amygdala. Studies carried out in Larry's laboratory showed that bilateral, selective amygdala damage did not affect performance on the DNMS task (Zola‐Morgan, Squire, and Amaral 1989a). This finding was consistent with findings from Mort's lab, so on its own did not influence the debate. What did influence Stuart and Larry, however, was their finding that selective amygdala damage, when added to (aspiration) hippocampal damage, did not add to impairments produced by aspiration lesions of the hippocampus (Zola‐Morgan, Squire, and Amaral 1989a). In addition, a test of the effects of combined damage to the hippocampus and perirhinal cortex produced a severe impairment on the DNMS task (Zola‐Morgan et al. 1993). These findings, taken together, cemented their view that the hippocampus, together with neighboring structures in the MTL—specifically the entorhinal cortex, perirhinal cortex and parahippocampal cortex—comprised an MTL memory system (Squire and Zola‐Morgan 1991).

In a highly influential review (Squire and Zola‐Morgan 1991), Larry and Stuart described the structures they considered necessary for establishing new declarative memories, the type of memory lost by H.M. In short, the review discussed data from studies in rats, monkeys and humans to make the case for the role of the MTL in declarative memory, building on the neuroanatomical connections of the region to make inferences about function. In their view, they had solved the puzzle of H.M. This soon led to the emergence of the main tenets of a doctrine called the MTL memory system: (1) the four cortical structures work together as a system in the service of declarative memory (Squire and Zola‐Morgan 1991); (2) the greater the damage to MTL structures, the greater the memory impairment (Zola‐Morgan, Squire, and Ramus 1994), more‐or‐less regardless of the precise location of the damage; (3) the MTL subserves memory not perception (Buffalo et al. 1999, 2000); and (4) the MTL subserves declarative memory not procedural memory (Malamut, Saunders, and Mishkin 1984; Squire 1992; Zola‐Morgan and Squire 1984), which Mort and Larry both called habits and consigned to the basal ganglia (Fernandez‐Ruiz et al. 2001; Knowlton, Mangels, and Squire 1996). What had started with a circumscribed question about the neural substrates of whatever is measured by the DNMS task had been hijacked to cover all declarative memory: the memory for facts and events in humans, with all its implications about consciousness. Mort, too, viewed the DNMS task as a measure of declarative memory, although he preferred the proxy term ‘cognitive memory’, which could be applied to humans and animals without needing to address the problematic concept of consciousness. I return to this topic in the final section of this article.

## 5 Recent Object Recognition Memory Studies

One addendum to the foregoing is a recent meta‐analysis re‐evaluating the effects of hippocampal and perirhinal cortex damage on object recognition memory and spatial memory (Waters, Basile, and Murray 2023). The studies included in this analysis were limited to those employing selective, excitotoxic lesions, which assessed memory using either the DNMS task or visual paired comparisons (VPC). VPC, also known as preferential viewing, relies on relative looking time as a measure of novelty. VPC has been used to study human infants and adults (Fantz 1964; Pascalis et al. 2004), and macaques (Buffalo 2024; Buffalo et al. 1999; Pascalis and Bachevalier 1999; Zola et al. 2000), and is typically considered to provide a measure of spontaneous object recognition memory (cf. (Basile, Waters, and Murray 2024)). Grouping studies by site of lesion (hippocampus, perirhinal cortex) and task (DNMS, VPC), separate meta‐analyses were conducted for object memory and spatial memory. Extending results of an earlier analysis carried out by Mark Baxter (Baxter and Murray 2001), one meta‐analysis indicated that impairments on tests of visual item recognition were larger after lesions of perirhinal cortex than after lesions of the hippocampus. A separate meta‐analysis showed that performance on tests of spatial navigation memory was severely impaired by lesions of the hippocampus. Interestingly, object memory impairments were task dependent; VPC and DNMS were differentially reliant on the hippocampus. VPC produced greater impairments following hippocampal damage and DNMS yielded greater impairments following perirhinal cortex damage (Waters, Basile, and Murray 2023). In retrospect, it seems likely that the use of VPC to measure object recognition may have contributed to conflicting reports of hippocampal involvement in object recognition, perhaps due to the influence of nonmnemonic factors on VPC performance (Basile, Waters, and Murray 2024).

## 6 The Moral of the Story

The moral of the story is to do the proper control experiments even when it is possible to make a big splash without them. At least initially, the results of our long‐delayed “control” experiments were completely unexpected, and we undertook them with little expectation that it would do anything other than confirm prevailing views about the MTL memory system. By doggedly pursuing a bedrock principal of experimental psychology, we obtained findings that led all that followed. Once we removed the perirhinal cortex completely, along with the entorhinal cortex, the monkeys had a profound impairment on the DNMS task: one approximately as large as the one Mort observed after what he called A + H lesions (Figure 2). As was instantly apparent, this finding sounded the death knell for the A + H/DNMS monkey model of human amnesia, and it did not bode well for the prevailing view about the so‐called MTL memory system either. This is in part because neither the amygdala nor the hippocampus is necessary for normal performance of the DNMS task, as our selective excitotoxic A + H lesions later confirmed. Furthermore, the DNMS task does not measure the kinds of memory that H.M. lost after his surgery. Among the consequences of our control experiments were projects directed to understanding the contribution of the amygdala to behavior (Izquierdo and Murray 2004; Malkova, Gaffan, and Murray 1997), collaborative projects with Tim Bussey and Lisa Saksida on the function of the perirhinal cortex (Bussey, Saksida, and Murray 2003; Murray, Bussey and Saksida 2007; Saksida et al. 2007), projects with Rob Hampton to evaluate hippocampal contributions to navigation (Hampton, Hampstead, and Murray 2004), and with Kim Graham and her colleagues on the differential functions of the hippocampus and other parts of what's called the MTL in humans (Barense et al. 2005; Lee et al. 2005). In addition, it spurred the use of novel paradigms to study hippocampal function in monkeys, some exploring the links between the activity of neurons in the hippocampus and entorhinal cortex, exploratory eye movements, navigation, and memory (Buffalo 2024; Rolls 2024), and others exploring the neural substrates of object‐in‐place scene memory (Gaffan 1994; Parker and Gaffan 1997).

Overturning the A + H monkey model of amnesia also spurred us to consider memory and representational systems more broadly and at book length (Murray et al. 2020; Murray, Wise, and Graham 2017), as mentioned earlier. Based on an evolutionary perspective, and drawing on many studies by friends and colleagues (Bussey and Saksida 2007; Gaffan 2002; Graham, Barense, and Lee 2010; Murray and Wise 2012), we developed the evolutionary accretion model of memory (Murray, Wise, and Graham 2017). In brief, the model proposes that memory systems evolved at various times in the distant past, as specific ancestors adapted to a new way of life, usually during major evolutionary transitions. Thus, as new representations evolved they were added (or accreted) to already existing systems. In contrast to the MTL memory system model, the evolutionary accretion model holds that: (i) all cortical areas have memory functions, which depend on their specialized representations; (ii) all memory systems depend on both the cerebral cortex and basal ganglia, connected in recurrent loops; (iii) in part because these loops include the hippocampus and other MTL areas, the idea that four cortical areas in the MTL have one function (explicit memory) whereas the basal ganglia has a different function (habits) can be rejected; and (iv) no single memory system accounts for explicit memory, which instead is an emergent property of interacting memory systems.

None of which would have happened without a relentless effort to get control experiments right.

## Data Availability

This article is a commentary; no new data are included.

## Endnotes

^1^
MRI machines became commercially available in the 1980s, and received approval by the Food and Drug Administration for clinical use in 1984. According to the US National Science Foundation, ‘By 1988, MRI had achieved significant penetration of its primary market: Clinical medicine.’
